# Characterization of the Chronic Risk and Hazard of Hazardous Air Pollutants in the United States Using Ambient Monitoring Data

**DOI:** 10.1289/ehp.11861

**Published:** 2009-01-09

**Authors:** Michael C. McCarthy, Theresa E. O’Brien, Jessica G. Charrier, Hilary R. Hafner

**Affiliations:** Sonoma Technology, Inc., Petaluma, California, USA

**Keywords:** air quality, air toxics, ambient air quality, hazardous air pollutants, risk screening

## Abstract

**Background:**

Ambient measurements of hazardous air pollutants (air toxics) have been used to validate model-predicted concentrations of air toxics but have not been used to perform risk screening at the national level.

**Objectives:**

We used ambient concentrations of routinely measured air toxics to determine the relative importance of individual air toxics for chronic cancer and noncancer exposures.

**Methods:**

We compiled 3-year averages for ambient measurement of air toxics collected at monitoring locations in the United States from 2003 through 2005. We then used national distributions of risk-weighted concentrations to identify the air toxics of most concern.

**Results:**

Concentrations of benzene, carbon tetrachloride, arsenic, 1,3-butadiene, and acetaldehyde were above the 10^−6^ cancer risk level at most sites nationally with a high degree of confidence. Concentrations of tetrachloroethylene, ethylene oxide, acrylonitrile, and 1,4-dichlorobenzene were also often greater than the 10^−6^ cancer risk level, but we have less confidence in the estimated risk associated with these pollutants. Formaldehyde and chromium VI concentrations were either above or below the 10^−6^ cancer risk level, depending on the choice of agency-recommended 10^−6^ level. The method detection limits of eight additional pollutants were too high to rule out that concentrations were above the 10^−6^ cancer risk level. Concentrations of 52 compounds compared with chronic noncancer benchmarks indicated that only acrolein concentrations were greater than the noncancer reference concentration at most monitoring sites.

**Conclusions:**

Most pollutants with national site-level averages greater than health benchmarks were also pollutants of concern identified in modeled national-scale risk assessments. Current monitoring networks need more sensitive ambient measurement techniques to better characterize the air toxics problem in the United States.

The U.S. Clean Air Act Amendments of 1990 regulate 187 hazardous air pollutants (air toxics) that are associated with a wide variety of adverse health effects, including cancer, neurologic effects, reproductive effects, and developmental effects [U.S. Environmental Protection Agency (EPA) 2000]. The list of air toxics includes volatile organic compounds (VOCs), metals in particulate matter (PM), and semi-volatile organic compounds with significant differences in chemical and physical characteristics. Likewise, a wide variety of anthropogenic sources such as automobiles, commercial and retail entities, and industrial sources emit air toxics. Air toxics may also be emitted by geogenic sources (e.g., volcanoes) or from biogenic sources (e.g., methyl chloride from coastal salt marshes). Removal rates of air toxics also vary significantly, with residence times ranging from a few minutes to decades. Based on these emissions and removal characteristics, significant differences in air toxics concentrations are expected across the United States.

Cancer and noncancer health impacts associated with environmental exposures to air toxics cannot be directly isolated and measured. However, scientists have developed risk assessment methodologies to estimate environmental health risks (National Research Council 1983). Hazard identification consists of identifying contaminants that may pose human health hazards at environmentally relevant concentrations and qualitatively describing the potential impacts on human health. Dose–response assessment characterizes the relationship between exposure to a pollutant and resultant health effects. In this study, we focused on the risk characterization step of risk assessment. Consequently, these results are intended to focus further measurement and assessment efforts.

Risk assessments in numerous studies have been performed either on limited subsets of air toxics or on the entire suite of air toxics in the United States (Caldwell et al. 1998; Loh et al. 2007; U.S. EPA 2002, U.S. EPA 2006a; Woodruff et al. 1998; Wu and Pratt 2001). Most of these studies have used the Assessment System for Population Exposure Nationwide (ASPEN) model to predict outdoor concentrations of air toxics (Caldwell et al. 1998; Loh et al. 2007; Rosenbaum et al. 1999; U.S. EPA 2002, U.S. EPA 2006a; Woodruff et al. 1998).

Although the ASPEN model provides predictions by census tract for the entire United States, it relies on the accuracy of the National Emissions Inventory, along with estimated background concentrations, to predict ambient concentrations. Comparisons of model predictions with ambient concentrations have shown that the ASPEN model underestimates most HAP concentrations, some by more than a factor of 2 (U.S. EPA 2006a). In particular, the model systematically under-predicts average metals concentrations. Model-predicted concentrations that are too low will result in underestimates of risks to human health, with subsequent implications for public policy development.

Ambient measurements were used as part of personal exposure studies (Kwon et al. 2006; Payne-Sturges et al. 2004). Annual mean concentrations at sites in the Los Angeles air basin were used in the Multiple Air Toxics Exposure Study to estimate risk associated with ambient concentrations (South Coast Air Quality Management District 2000). In addition, Loh et al. (2007) referenced a handful of localized risk monitoring studies over disparate time periods and locations to estimate the distribution of concentrations nationally.

In this study, we compared distributions of ambient concentrations for all measured air toxics available from 2003 through 2005 with U.S. EPA–recommended chronic health benchmarks (U.S. EPA 2006a). We compiled ambient monitoring data from hundreds of monitoring locations to create the most complete data set of air toxics measurements. The results of this study allow us to compare the national distributions of air toxics ambient concentrations for both cancer and noncancer chronic exposures across the United States.

## Methods

The U.S. EPA maintains a national repository of ambient air quality data collected by local, state, tribal, and federal entities called the Air Quality System (AQS) (U.S. EPA 2006b). We acquired data from the AQS for the period 2003 through 2005 and augmented them with data from the Interagency Monitoring of Protected Visual Environment for metals in PM with aerodynamic diameter ≤ 2.5 μm (PM_2.5_) (which are typically not in the AQS) collected at national park monitoring locations for the same period (Malm et al. 2004). Data validation, screening, and averaging (i.e., daily, quarterly, annual, and interannual) were performed before the data were used for analysis in this study. The screening and validation of this database are described in detail elsewhere (McCarthy et al. 2007). We include a brief summary here.

Toxic pollutant concentrations in the database were typically available as 24-hr duration samples collected at daily, 1-in-3-day, 1-in-6-day, or 1-in-12-day frequencies. We averaged these 24-hr samples to quarterly and annual mean concentrations. For a given pollutant, site, and year, we calculated quarterly mean concentrations based on one of two completeness criteria. If the typical sampling frequency was determined to fit one of the patterns (daily, 1-in-3-day, 1-in-6-day, or 1-in-12-day), we calculated quarterly averages based on a 75% completeness criterion (e.g., 12 of 15 samples per quarter would be required for 1-in-6-day sampling). For the second criterion, we could not assign the frequency of a given pollutant at a site based on the samples available. In this second case, we required at least six daily average concentrations, representing 75% completeness for a 1-in-12-day sampling schedule, for a calendar quarter. In addition, the first and last of these samples had to be at least 58 days apart to ensure representativeness across the 3-month time period.

We then used valid quarterly mean concentrations to generate annual means with an additional 75% completeness criterion (i.e., we required three of four quarters for a year). Therefore, annual mean concentrations were not unduly skewed by seasonal variations in concentrations (McCarthy et al. 2007).

We averaged valid annual mean concentrations at individual locations from 2003 through 2005 to create “site averages.” Site averages were considered valid if one, two, or three annual averages were available; this validity test maximized the number of site averages available for analysis. Although we recognize that this approach may introduce a confounding temporal factor into the analysis, the step was necessary to include as many data of reasonable quality as possible in the analysis; choosing only a single year of data would minimize the number of available sites. We chose the 2003–2005 time period for two reasons: *a*) These concentrations were the most recent available and therefore most likely reflected current concentrations and variability, and *b*) this time period provided the most available data (i.e., both geographically and by number of sites).

**Figure 1:**
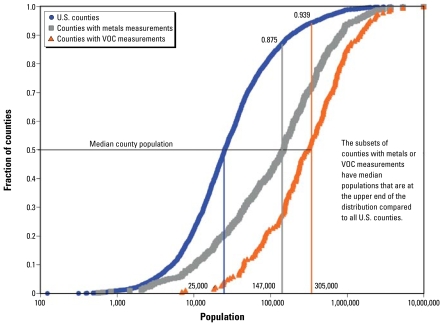
Cumulative distribution function illustrating the differences in distributions of county VOC and PM metal monitoring locations and U.S. counties. The subsets of counties with air toxics measurement sites were significantly more urbanized and populated compared with U.S. counties in general.

Figure 1 illustrates the distribution of monitoring sites in the United States relative to the distribution of county populations and shows the significant overrepresentation of urban counties among the monitoring population relative to the distribution of all counties in the United States. This distribution is sensible for exposure monitoring, because urban counties represent the largest fraction of the population and also the highest emission rates for most air toxics. However, the following results should be understood in the context of the predominantly urban monitoring network, which will skew the concentration distributions toward higher values relative to the true national distribution.

### Values below the method detection limit

In this study, many of the measurements were at levels below the method detection limit (MDL). Across all air toxics, about 60% of reported measurements in the data set were below the MDL. Data below MDL are reported to AQS in a variety of ways, including values of zero, MDL, MDL/2, MDL/3, or the measured value. Using reported values can introduce systematic jurisdictional biases for values below MDL. However, the large fraction of values below MDL did not preclude their use in this analysis. When even a small fraction of reported data exceeds the MDL, techniques are available to calculate annual averages with reasonably small uncertainties, such as substitution, Kaplan–Meier, and regression on order statistics. We chose to use a simple MDL/2 substitution technique to calculate annual averages because it was much simpler to implement and gave results roughly equivalent to more sophisticated statistical treatments (Antweiler and Taylor 2008). We substituted MDL/2 for individual measurements below the MDL when we compiled quarterly averages. We tracked counts of substituted values throughout the process. In unpublished work, we found that as the percentage of measurements below MDL exceeds 85%, the relative bias in concentrations becomes as large as the estimated concentration. This result is similar to a cutoff of 70% found by Antweiler and Taylor (2008). Given that many pollutants have lognormal distributions of concentrations in the atmosphere (Seinfeld and Pandis 1998), MDL/2 substitution will usually result in overestimating mean concentrations when > 85% of measurements are below the MDL, so we consider it an upper limit in these cases. For additional details showing the results of our MDL substitution analysis, see Supplemental Material and Supplemental Figure 1 (http://www.ehponline.org/members/2009/11861/suppl.pdf).

### Approaches for comparing hazard between pollutants

We used three approaches to characterize the nature and extent of the HAP problem using ambient concentrations of pollutants in a risk context. In the first method, we weighted national concentration distributions using chronic health benchmarks to identify pollutants with concentrations typically greater than levels of concern. We used the U.S. EPA Office of Air Quality Planning and Standards (OAQPS) list of recommended chronic health benchmarks for this analysis (U.S. EPA 2007). We multiplied the unit risk estimate values by site average concentrations to create risk-weighted concentrations for each location. We divided site averages by noncancer reference concentrations to create hazard quotients for comparison. Note that uncertainties in the values of the chronic risk and hazard levels of concern can range across many orders of magnitude and are an active area of research. Using alternate values (California Air Resources Board 2004) for these chronic benchmarks will result in substantial differences in results for some pollutants. Formaldehyde and chromium VI both have significant differences in benchmarks, and results for alternative benchmarks are also shown.

In the second approach, we compared individual site averages with health benchmarks to determine the fraction of sites reporting values above chronic health benchmarks. We used three categories: above the benchmark, below the benchmark, or indeterminate. We used the third category when 85% of site average measurements were below the MDL and the benchmark was below the MDL. We categorized sites and tabulated the results to illustrate the pollutants of concern.

In the third approach, we displayed pollutants on maps to provide visual examination of the spatial variations in risk-weighted concentrations. Graphically displayed risk-weighted concentrations illustrate the relative concentrations for each site in the United States. These color-coded maps indicate breakpoints in the risk values and illustrate where data are unreliable for quantitatively assessing concentrations. Because a large number of air toxics were screened, these maps are available online as Supplemental Material, Figures 2–176 (http://www.ehponline.org/members/2009/11861/suppl.pdf).

## Results

The database of air toxics is heterogeneous in its national coverage of air toxics. We investigated 65 air toxics with health benchmarks and monitoring data at a minimum of 10 sites. Data for some pollutants were available from > 500 monitoring sites, whereas monitoring data for other pollutants were not available at all. Pollutants excluded from this study because of lack of monitoring data or health benchmarks may contribute substantially to national risk or hazard, but are not discussed here because of the lack of monitoring or toxicity information.

### National risk distributions

**Figure 2:**
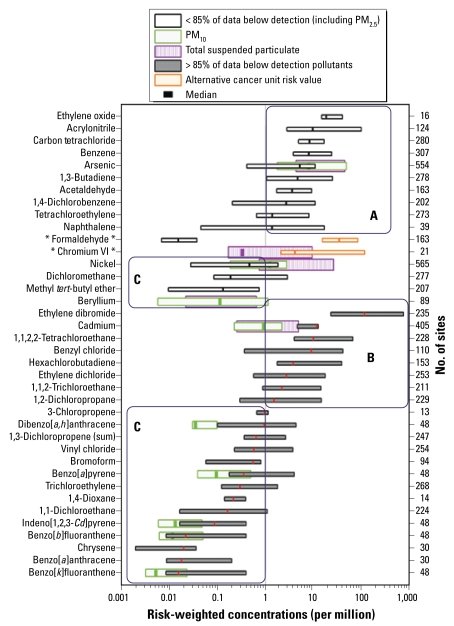
National distributions of cancer risk–weighted concentrations. Each bar shows the 5th to 95th percentile risk-weighted concentration range for a given pollutant. The lines in the middle of the bars denote median concentrations. Overlapping bars for PM metals display the three size fractions. Concentrations are weighted by the U.S. EPA OAQPS-recommended chronic unit risk estimates. For descriptions of the lettered boxes surrounding the groups of pollutants, see “Results.” Pollutants with alternative unit risk estimates described in the text are listed with asterisks around their names.

Figure 2 shows the site-level distribution of cancer risk–weighted concentration ranges for pollutants at all sites across the United States, along with the 5th, 50th (median), and 95th percentiles for each distribution. The right *y*-axis displays the number of monitoring sites used to create each distribution. The multiple overlaid boxes indicate particulate metals measured in multiple size fractions. Values > 1 reflect concentrations greater than the 10^−6^ risk level and identify pollutants of potential concern. Pollutants are organized by their relative median risk-weighted concentrations and sorted into two groups. The pollutants at the top of the figure are those for which at least 15% of the total number of measurements reported nationally from 2003 through 2005 are above MDLs; the pollutants at the bottom are those for which < 15% of their concentrations are above their respective MDLs. Pollutants are grouped into categories A, B, and C. Pollutants in category A are those for which most sites report risk-weighted concentrations above the 10^−6^ benchmark. Pollutants in category B are those for which 85% of the data nationally are below detection limits, but detection limits are above the 10^−6^ benchmark. These pollutants potentially have concentrations above the 10^−6^ level, but that cannot be determined using the available monitoring data. In contrast, pollutants in group C are those for which most monitoring sites report concentrations below the 10^−6^ benchmark. Group C pollutants include those that are well monitored and poorly monitored. In both cases, we are certain that national median concentrations are lower than the 10^−6^ level.

Pollutants in category A are those for which measurements are mostly above the 10^−6^ benchmark. The group includes ethylene oxide, acrylonitrile, benzene, carbon tetrachloride, arsenic, 1,3-butadiene, 1,4-dichlorobenzene, acetaldehyde, naphthalene, tetrachloroethylene, and the larger PM size fractions of nickel. Some pollutants, such as arsenic, benzene, and carbon tetrachloride, were monitored at hundreds of locations. In contrast, pollutants such as ethylene oxide and naphthalene were monitored at only 16 and 39 sites, respectively. Pollutants measured at fewer locations may be a poorer representation of the national distribution. Of note, measurements of many pollutants in category A are above levels of concern at all locations. In contrast, concentrations of pollutants such as 1,4-dichlorobenzene, tetrachloroethylene, and naphthalene are below the level of concern at a significant number of locations and have lower risk-weighted concentrations in general. Note that formaldehyde and chromium VI are displayed as having two national risk distributions. Formaldehyde measurements were below the level of concern at all sites in this study using the OAQPS benchmark and above the level of concern at all sites using the Integrated Risk Information System (IRIS) cancer benchmark (U.S. EPA 2008). Similarly, chromium VI levels were mostly below the benchmark using the OAQPS/IRIS benchmark and above the benchmark using the California Environmental Protection Agency (CalEPA) cancer benchmark (California Air Resources Board 2004). We show the results of both in Figure 1, illustrating how the differences in benchmarks can be paramount for interpreting the potential health outcomes.

Pollutants in category B have concentrations that potentially exceed the 10^−6^ benchmark. However, these pollutant levels are below MDLs more than 85% of the time; therefore, concentration distributions shown in Figure 2 reflect the distribution of typical MDL/2 values, rather than actual ambient concentrations. Pollutants in group B include ethylene dibromide, cadmium, 1,1,2,2-tetrachloroethane, benzyl chloride, hexachlorobutadiene, ethylene dichloride, 1,1,2-trichloroethane, and 1,2-dichloropropane. The concentration distributions shown are likely to be qualitative upper-limit estimates of risk-weighted concentrations for these pollutants. True ambient concentration distributions may be below the 10^−6^ level of concern, but this conclusion cannot be determined with confidence using the monitoring data alone. Lower MDLs would help reduce the uncertainty in ambient risk estimates for these pollutants. Modeling concentrations of these pollutants may be more reliable than using ambient measurements that are almost always below MDL.

Pollutants in group C are those for which levels are below the 10^−6^ level of concern at more than half the monitoring sites around the country. Group C pollutants include those with levels usually above MDL, such as dichloromethane, and with levels usually below the MDL, such as vinyl chloride and trichloroethylene. Although the actual concentrations may be poorly quantified because ambient concentrations are below MDL, it is sufficient to say that median national concentrations are below the 10^−6^ benchmark.

**Figure 3:**
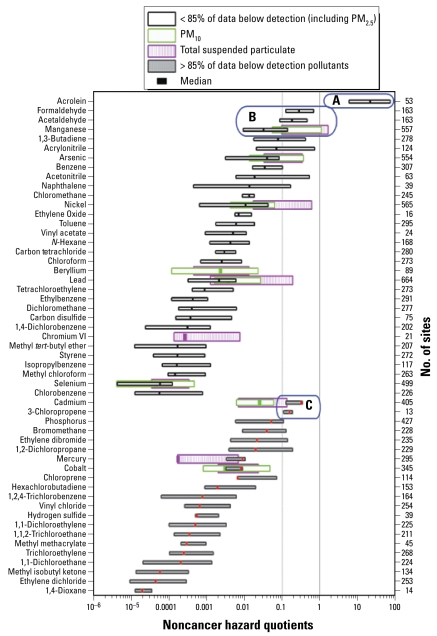
National distributions of noncancer hazard quotients. Each bar shows the 5th to 95th percentile risk-weighted concentration range for a given pollutant. The lines in the middle of the bars denote median concentrations. Overlapping bars for PM metals display the three size fractions. Concentrations are weighted by the U.S. EPA OAQPS-recommended chronic reference concentrations. For descriptions of the lettered boxes surrounding the groups of pollutants, see “Results.”

Figure 3 shows the 5th, 50th (median), and 95th percentile noncancer hazard quotient distributions for pollutants across the United States. The figure uses conventions similar to those shown in Figure 2; the primary difference lies in the noncancer hazard quotients. Values > 1 are above the noncancer reference concentration; values > 0.1 are considered to be of potential concern. Three specific pollutant groups are labeled A, B, and C. Acrolein is the only pollutant in group A; its national distribution of concentrations is greater than the chronic noncancer reference concentration. Pollutants in group B have a hazard quotient between 0.1 and 1.0 at most monitoring sites. Formaldehyde, acetaldehyde, and manganese [both total suspended particulates (TSP) and PM_10_ (PM with aerodynamic diameter ≤ 10μm) size fractions] are in this category. Pollutants in group C are those for which concentrations cannot be determined to be below the 0.1 level of concern. Cadmium PM_2.5_ and 3-chloropropene are both inadequately measured and cannot be proven to be below the level of concern. All other pollutants are typically below levels of potential concern at most sites. Figure 3 clearly shows that the noncancer hazard from acrolein is an order of magnitude greater than that of any other pollutant.

### Categorical comparison

A second method of examining the national data is to look at the summary statistics of individual sites relative to the chronic health benchmarks. We compared site-average concentrations from 2003 through 2005 with chronic health benchmarks at an individual site level and tabulated the results. We classified individual sites in one of three categories: above the benchmark, below the benchmark, and indeterminate. The “indeterminate” category includes all those sites where the average concentration is below the MDL and the health benchmark is below the MDL. In these cases, we cannot determine with confidence whether the average concentration is above or below the level of concern.

Table 1 catalogs the pollutants whose concentrations are above or potentially above the 10^−6^ benchmark for cancer risk at more than 50% of the locations. The third column lists the number of monitors with both > 15% of measurements above the MDL and mean concentrations above the 10^−6^ benchmark. The fourth column lists the number of monitors with < 15% of measurements above MDL and an MDL above the 10^−6^ benchmark. The pollutants benzene, acetaldehyde, carbon tetrachloride, arsenic, and 1,3-butadiene are measured at > 100 locations nationally, and at least 50% of those locations report average concentrations exceeding the benchmark. Among other pollutants listed in Table 1, we have lower confidence in reaching conclusions about national concentrations relative to the 10^−6^ benchmark. For example, ethylene dibromide is measured at hundreds of monitoring locations nationally, but the reported concentrations are below the MDL at 97% of sites and cannot be used to quantitatively assess whether national concentrations are above or below the 10^−6^ benchmark. Additionally, some pollutants such as ethylene oxide are measured at too few sites to assume a nationally representative pattern. More sensitive monitoring techniques or additional monitoring locations are needed to improve our confidence in assessing the risk posed by these pollutants.

Table 2 catalogs the pollutants for which at least 1% of their individual site measurements are at or above the noncancer reference concentration level. Of note, only acrolein values are above the noncancer reference concentration at a large fraction of monitoring sites. No other pollutant has concentrations above levels of concern at > 15% of monitoring sites. This significant difference reinforces the data in Figure 3 indicating that acrolein is the most important pollutant to consider for chronic noncancer health effects.

### Spatial variability

**Figure 4:**
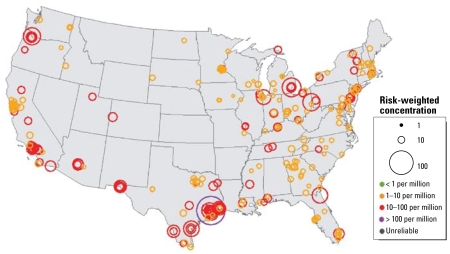
Risk-weighted concentrations of benzene (per million) at coterminous U.S. sites between 2003 and 2005. Circled areas indicate the magnitude of risk associated with each site.

**Figure 5:**
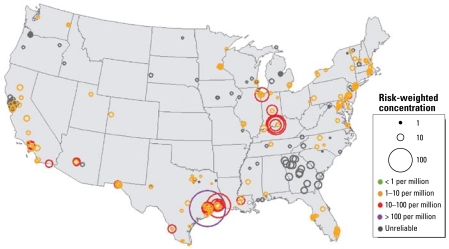
Risk-weighted concentrations of 1,3-butadiene (per million) at coterminous U.S. sites between 2003 and 2005. Circled areas indicate the magnitude of risk associated with each site. Gray circles indicate sites with poorly characterized concentrations and should be considered as upper-limit estimates.

Figures 4 and 5 illustrate cancer risk–weighted concentrations of benzene and 1,3-butadiene, respectively, at individual sites across the United States. The circle sizes indicate the relative value of the risk-weighted concentration (e.g., larger circles indicate higher values). Circles are centered on the locations of the monitoring sites they represent. Color coding indicates key levels of risk by factors of 10; gray indicates that the concentration data are unreliable. In Figure 4, risk-weighted concentrations of benzene are primarily orange (1–10 per million) and red (10–100 per million). Sites, cities, and regions with particularly high or low concentrations can be identified visually. Concentrations of benzene are highest in many of the large urban centers, although there are many exceptions. Overall, the range of risk-weighted concentrations is always greater than the level of concern and clearly indicates that benzene concentrations are a national problem.

Figure 5 shows the risk-weighted concentrations of 1,3-butadiene. Overall, most sites display lower risk-weighted concentrations of 1,3-butadiene than benzene (comparing Figures 4 and 5). The locations of highest concentrations of 1,3-butadiene are Louisville, Kentucky, and Houston, Texas, both of which have large industrial sources of 1,3-butadiene. In addition, a large number of sites had > 85% of their average concentrations below the MDL (gray circles). These concentrations should be considered unreliable indicators of the true risk-weighted value. However, these unreliable sites can still be useful by indicating an approximate upper limit of risk values at these sites within a factor of 2. Given typical lognormal atmospheric distributions, the magnitudes of the circles are likely to be an upper limit for risk-weighted concentrations and can indicate jurisdictions that may need to lower their MDL values to accurately measure concentrations at levels of concern.

Additional maps of all pollutants, for both chronic cancer risk and noncancer risk, are available in Supplemental Material, Figures 2–176 (http://www.ehponline.org/members/2009/11861/suppl.pdf).

## Discussion

Ambient concentrations of multiple air toxics routinely exceed the health benchmark. We are highly confident that concentrations for six pollutants are above levels of concern at most monitoring locations. Benzene, 1,3-butadiene, carbon tetrachloride, acetaldehyde, and arsenic are all monitored at hundreds of locations where concentrations exceed the level of concern for cancer risk, whereas acrolein concentrations exceeded the noncancer reference concentration at a minimum of 77% of its monitoring locations. Modeling studies have also listed these pollutants among the most important air toxics for human health. For example, Woodruff et al. (1998) and the U.S. EPA (2002 U.S. EPA (2006a) identified each of these toxics as being above the health benchmarks.

We have less confidence in assessing typical national levels of risk using the ambient measurements of ethylene oxide, acrylonitrile, 1,4-dichlorobenzene, naphthalene, and larger PM size fractions of nickel. These pollutants are monitored at fewer locations and/or have more measurements below the MDL. Comparing these pollutants with those in other risk assessments studies can provide additional insight into the reliability of the ambient measurements for screening risk.

The median risk-weighted concentration of ethylene oxide was the highest of all ambient pollutants measured with data above MDL. Ethylene oxide was considered a low risk in the model assessment of California by Kyle et al. (2001), and was estimated by the U.S. EPA (2006a) to have higher than 10^−5^ risk to fewer than 500,000 people. Ambient levels of ethylene oxide were measured only in the New England states, at 16 locations. Given the high risk-weighted concentrations measured there, additional measurements are needed in other areas to determine whether ethylene oxide is a national problem.

Risk-weighted concentrations of acrylonitrile were very high in many regions of the country and exceeded 10^−6^ at all sites with concentrations above the MDL. Acrylonitrile was identified by Woodruff et al. (1998) as being above the 10^−6^ cancer benchmark in 25% of U.S. census tracts, and the National Air Toxics Assessment (NATA) 1999 (U.S. EPA 2006a) estimated that fewer than 18 million people were exposed to acrylonitrile at concentrations above the 10^−6^ benchmark. Acrylonitrile was considered to have a low probability of being a health concern by the model assessment of Kyle et al. (2001). Monitoring methods that vary among jurisdictions and high MDLs relative to the cancer benchmark are limitations for performing risk screening using ambient data for this pollutant. However, given the high risk-weighted concentrations identified in this study at the 124 monitoring sites nationally, acrylonitrile should be considered a priority pollutant for additional scrutiny, such as measurement technique intercomparisons and the addition of monitors.

Risk-weighted concentrations of 1,4-dichlorobenzene were higher than the 10^−6^ level at one-third of sites nationally. However, the MDLs at 51% of sites were too high to determine whether concentrations were above the health benchmark. With up to 84% of sites reporting values above the health benchmark, better detection limits are needed to quantify 1,4-dichlorobenzene. NATA 1999 (U.S. EPA 2006a) estimated that about 42 million people in the United States were exposed to concentrations above the 10^−6^ cancer benchmark, and a recent hybrid model-monitor exposure assessment by Loh et al. (2007) characterized total risk from 1,4-dichlorobenzene as being above the 10^−6^ benchmark (although a significant portion of this exposure ranking was attributed to indoor sources of 1,4-dichlorobenzene).

Few sites monitored naphthalene, which, when well measured, usually had concentrations above the 10^−6^ level of concern. NATA 1999 (U.S. EPA 2006a) estimated about 180 million people exposed to naphthalene concentrations above the 10^−6^ benchmark level, and naphthalene concentrations were also estimated to be above the 10^−6^ benchmark by Loh et al. (2007). However, the contribution to exposure reported by Loh et al. (2007) was attributed mostly to indoor sources of naphthalene. Additional naphthalene monitoring sites are warranted to characterize the national risk from naphthalene based on these observations and previous results.

Nickel measurements in multiple-size fractions reported in this analysis were available from > 500 monitoring sites. Overall, nickel concentrations in the larger size fractions were likely to be above the health benchmark for nickel subsulfide, which may be a substantial fraction of total nickel emitted. However, fewer than 100 monitoring sites measured these larger size fractions. More than 400 monitoring sites measured PM_2.5_. Risk-weighted concentrations in this smaller size fraction were only infrequently above the 10^−6^ health benchmark. Woodruff et al. (1998) reported nickel concentrations above the 10^−6^ level at fewer than half of all census tracts, whereas NATA 1999 (U.S. EPA 2006a) estimated that fewer than 5 million people were exposed to concentrations above the 10^−6^ benchmark. The spatial variability of the available monitoring measurements is sufficient to state that nickel concentrations are primarily above the 10^−6^ level of concern in the industrial Midwest and the Northeast corridor [see Supplemental Material, Figures 2–176 (http://www.ehponline.org/members/2009/11861/suppl.pdf)].

Some pollutant concentrations could not be quantified well enough using ambient measurements to draw definite conclusions from this screening analysis. Pollutants that potentially have risk-weighted concentrations above the 10^−6^ benchmark include ethylene dibromide, cadmium, 1,1,2,2-tetrachloroethane, benzyl chloride, hexachlorobutadiene, 1,1,2-trichloroethane, and 1,2-dichloropropane. These pollutants have MDLs higher than the cancer benchmark and concentrations below MDLs > 85% of the time. Therefore, the concentration distributions only illustrate the upper ranges of risk associated with the compounds. Results from Woodruff et al. (1998) show that ethylene dibromide was above the health benchmark at more than half of all census tracts, and cadmium was above at > 25% of census tracts. Fewer than 25% of census tracts show both 1,1,2-trichloroethane and 1,1,2,2-tetrachloroethane above their respective cancer benchmarks. NATA 1999 indicated that cadmium levels were above the 10^−6^ level for only about 6 million people, whereas ethylene dibromide and 1,1,2,2-tetrachloroethane levels were above the 10^−6^ level for about 250 million people (U.S. EPA 2006a). No other pollutants in this study were mentioned as possible risk contributors in NATA 1999. Given the possible risk levels associated with these poorly quantified pollutants at current monitoring MDLs, sampling and analytical methods capable of quantifying concentrations for these pollutants at levels of concern for human health would be a significant improvement.

Some pollutants identified by ambient risk-weighted concentrations considered unlikely to be at concentrations of national concern include dichloromethane, beryllium, vinyl chloride, trichloroethylene, and most individual polycyclic aromatic hydrocarbons. Many of the results from this study are different from those reported in previous risk assessments.

Formaldehyde measurements were below the level of concern at all sites in this study using the OAQPS benchmark and above the level of concern at all sites using the IRIS 10^−6^ cancer benchmark (Table 1). Formaldehyde has been listed as a key risk driver in other studies (e.g., Loh et al. 2007; Woodruff et al. 1998). The discrepancy here reflects the differences in the cancer benchmarks. The CalEPA unit risk value is in better agreement with the IRIS value.

Similarly, chromium VI levels were below the level of concern at most of the 21 sites where it was measured directly using the OAQPS/IRIS benchmark and above the level of concern at most sites using the CalEPA benchmark. The chromium VI screening is complicated by the underlying exposure measurements being based on an assumed fraction of total chromium existing in the hexavalent oxidation state. The two ranges provide a reasonable estimate of the potential sensitivity of this indicator, although the limited number of measurement sites is also a significant constraint on interpretation of this risk range. Additional measurements are needed to more accurately determine the national risk level of chromium VI.

Vinyl chloride concentrations were almost always below the level of the cancer benchmark and the MDL. In contrast, both Woodruff et al. (1998) and NATA 1999 (U.S. EPA 2006a) ranked vinyl chloride among the top contributors to cancer risk, although this was due to very high risk values in the top quartile of counties nationally, rather than median counties. Our finding is consistent with recent results from Loh et al. (2007), who determined that vinyl chloride is the smallest contributor to cancer risk among organics estimated to be responsible for > 87% of cumulative risk.

Among noncancer pollutants, our data show that only acrolein estimates are consistently above the level of concern at most monitoring locations. This conclusion is consistent with the findings of NATA 1999 (U.S. EPA 2006a) and Woodruff et al. (1998). Better quantification of risk associated with acrolein concentrations through additional risk assessment (Woodruff et al. 2007) and improved measurement techniques is warranted.

## Conclusions

We compared risk-weighted concentrations of ambient measurements of air toxics to identify pollutants of concern. Many key pollutants contributing to risk were in good agreement with other regional and national risk assessments. However, significant discrepancies among some pollutants illustrate that large uncertainties remain for quantifying concentrations of the air toxics using ambient measurements concentrations or models. Measurement methods capable of quantifying concentrations at levels of concern are needed to better estimate risk associated with the ambient concentrations of many pollutants. In addition, some pollutants are measured at too few locations to be considered representative of national concentrations.

## Figures and Tables

**Table 1:** Pollutants whose concentrations exceed or potentially exceed 10^−6^ cancer risk levels at > 50% of monitoring locations.

Pollutant	No. of locations	Percent locations with concentrations > 10^−6^ cancer risk^a^	Percent locations with concentrations potentially > 10^−6^ cancer risk^b^
Benzene	305	100	0
Formaldehyde	163	0 (100)^c^	0
Acetaldehyde	163	99	0
Chromium VI	21	10 (96)^c^	0 (4)^c^
Carbon tetrachloride	278	85	15
Ethylene oxide	16	81	19
1,3-Butadiene	276	70	30
Arsenic PM_2.5_	432	67	11
Arsenic PM_10_	37	59	38
Nickel PM_10_	35	57	9
Acrylonitrile	124	52	48
Arsenic TSP	82	40	60
Tetrachloroethylene	271	37	51
Cadmium PM_10_	36	36	22
Naphthalene	39	33	51
1,4-Dichlorobenzene	202	33	51
Nickel TSP	101	26	56
Cadmium TSP	105	15	57
Benzyl chloride	110	13	71
Hexachlorobutadiene	153	8	89
1,1,2,2-Tetrachloroethane	226	5	94
1,2-Dichloropropane	227	5	78
Vinyl chloride	252	4	70
Ethylene dichloride	251	4	95
1,1,2-Trichloroethane	211	3	96
Ethylene dibromide	233	3	97
Cadmium PM_2.5_	261	2	98
Dibenzo[*a*,*h*]anthracene	30	0	63

a Sites where < 85% of measurements were reported as below the MDL and the site average concentration was above the 10^−6^ cancer benchmark.

b Sites where > 85% of measurements were reported as below the MDL and the 10^−6^ cancer benchmark was below the MDL.

c Results change depending on the cancer benchmark value used (OAQPS or IRIS for formaldehyde, OAQPS or IRIS or CalEPA for chromium VI).

**Table 2:** Pollutants whose concentrations exceed or potentially exceed noncancer reference concentration levels at > 1% of monitoring locations.

Pollutant	No. of locations	Percent locations > reference concentration^a^	Percent locations potentially > reference concentration^b^
Acrolein	53	77	23
Manganese TSP	96	8	6
Manganese PM_10_	26	4	0
Acetonitrile	63	3	0
Formaldehyde	163	2	0
Acrylonitrile	124	2	0
1,3-Butadiene	276	1	0
Nickel TSP	101	1	6

a Sites where < 85% of measurements were reported as below the MDL and the site average concentration was above the reference concentration.

b Sites where > 85% of measurements were reported as below the MDL and the 10^−6^ reference concentration was below the MDL.
